# The role of neuroimmune and inflammation in pediatric uremia-induced neuropathy

**DOI:** 10.3389/fimmu.2022.1013562

**Published:** 2022-09-15

**Authors:** Linfeng Zhu, Guoqin Tong, Fan Yang, Yijun Zhao, Guangjie Chen

**Affiliations:** ^1^ Department of Urology, The Children’s Hospital, Zhejiang University School of Medicine, National Clinical Research Center for Child Health, Hangzhou, China; ^2^ Department of Neurology, The First People’s Hospital of XiaoShan District, Hangzhou, China

**Keywords:** uremia, neuroimmune, inflammation, oxidative stress, pediatric

## Abstract

Uremic neuropathy in children encompasses a wide range of central nervous system (CNS), peripheral nervous system (PNS), autonomic nervous system (ANS), and psychological abnormalities, which is associated with progressive renal dysfunction. Clinically, the diagnosis of uremic neuropathy in children is often made retrospectively when symptoms improve after dialysis or transplantation, due to there is no defining signs or laboratory and imaging findings. These neurological disorders consequently result in increased morbidity and mortality among children population, making uremia an urgent public health problem worldwide. In this review, we discuss the epidemiology, potential mechanisms, possible treatments, and the shortcomings of current research of uremic neuropathy in children. Mechanistically, the uremic neuropathy may be caused by retention of uremic solutes, increased oxidative stress, neurotransmitter imbalance, and disturbance of the blood-brain barrier (BBB). Neuroimmune, including the change of inflammatory factors and immune cells, may also play a crucial role in the progression of uremic neuropathy. Different from the invasive treatment of dialysis and kidney transplantation, intervention in neuroimmune and targeted anti-inflammatory therapy may provide a new insight for the treatment of uremia.

## 1 Introduction

Uremia, a term used to describe the presenting syndrome patients experience in end-stage renal disease (ESRD), is characteristic for a variety of clinical and metabolic disorders with progressive kidney failure, which usually caused by chronic kidney disease (CKD) or acute kidney injury (AKI) (1). It can cause the accumulation of organic waste products in plasma, referred to as uremic solutes or uremic toxins, which are not yet fully identified (2). Besides uremia toxins, oxidative stress and inflammation also persist in uremia. These risk factors may involve in generalized organ dysfunction and consequently result in a significant decrease in quality of life and increased mortality, which has made uremia rapidly become an urgent public health problem worldwide. Due to the disturbance of the internal environment, uremia can cause a series of complications. Generally, nervous system dysfunction is quite common in patients with uremia, knowns as uremic neuropathy. Patients with uremic neuropathy may experience insomnia, agitation, paranoia, cognitive impairment, and coma for weeks or months or even years, followed by exacerbation and ultimately a poor prognosis (3). It has been also reported that sleep disorders and restless legs syndrome are common in dialysis patients with neurological complications. The pathogenesis of uremic neuropathy remains unclear.

Children as a special population are gradually arousing concern among patients with uremia. Pediatric uremia differs from adult uremia in that the largest diagnosed group in children are associated with congenital abnormalities and inherited disorders, whereas glomerular and renal diseases in the context of diabetes are relatively rare. At present, the treatment of children with uremia is still mainly dialysis and kidney transplantation (4, 5). Additionally, since children are in a critical period of growth and development, the therapy of recombinant human growth hormone is required for the complications of growth retardation caused by uremia (6, 7). Further, uremia in children can also cause neurological complications that not only lead to substantial disability and mortality but also incite medical issues beyond childhood. It is generally known that the first year after birth is a critical period for brain development, with the proliferation of glial cells and the rapid formation of myelin sheaths (8). Therefore, any factor that interferes with these two processes could have a major impact on brain development. Multiple neurological and developmental abnormalities have been identified in children who develop chronic renal failure during the first year of life. These abnormalities include microcephaly, retardation, cerebral edema, recurrent seizures and ataxia (9–11). Moreover, children between 3 and 16 years old with chronic renal impairment are more likely to have hearing impairment, decreased hearing sensitivity (12). Children born by mothers with chronic kidney disease are more likely to develop to cerebral palsy and intellectual disability (13). This may suggest that the CNS of children is more susceptible to uremia. However, studies on the epidemiology and corresponding mechanisms of uremia-induced neuropathic disorders in children are scarce.

The aim of this review was to investigate the epidemiology, clinical features, underlying mechanism and possible treatments of the neuropathic disorders in pediatric uremia, in order to provide new insights for pediatricians.

## 2 Epidemiology of pediatric uremia

Epidemiological researches of uremia on growing children are scarce. Nonetheless, given that uremia is usually caused by CDK and AKI, the epidemiology of CDK and AKI indirectly reflects the prevalence of uremia. According to a report from the United States, the incidence of CDK in adults is approximately 11%, which is 50 times greater than that of ESRD (14). Besides, a review of 15-year admissions in Nigeria estimated the prevalence of severe pediatric CKD to be 15 cases per million (15). The average incidence of CDK in Kuwaiti children was 38.2 cases per million per year (16). The Swedish national survey reported a prevalence of advanced CKD was 21 cases per million per year (17). A population-based data from Italy reported a point prevalence of CKD of 74.7 cases per million per year (18). Taken together, compared with adults, the prevalence of CKD in children has been reported at 15-74.7 per million, while the incidence of AKI ranged from 37% to 51% (19). Such variation in data is likely to be influenced by geography, culture and the medical resources. Moreover, the prevalence of ESRD in children is less than 2% of the total ESRD population (20). Nonetheless, pediatric CKD is a progressive disease, and the mortality rate among children with ESRD who receive dialysis is 30 to 150 times higher than the general pediatric population (21). During 2012, the prevalence of ESRD in children treated with dialysis in a Brazilian cohort was 20 cases per million (22), while a nation-wide survey from China between the 2007 to 2012 showed that 45.9% children with ESRD were on dialysis (23). The 1-, 2-, and 5- year overall survival rates of children on dialysis were 96.9%, 94.5%, 89.5% respectively (24). Furthermore, uremic neuropathy is quite prevalent in children with CKD on continuous hemodialysis. The prevalence of uremic neuropathy in children is not available, however, a small sample research reported that it was approximately 22% (25). Thus, the diagnosis and treatment of pediatric CKD and AKI must emphasize early detection, primary prevention, and active management to avoid further progression to ESRD and uremia. A population-based epidemiology of uremia in children is urgently needed.

## 3 Uremic neuropathy

### 3.1 Background

Uremic neuropathy encompasses a wide range of neurological abnormalities associated with poor renal function. It can involve the central nervous system (CNS), peripheral nervous system (PNS), autonomic nervous system (ANS) as well as psychological abnormalities (**Table 1**). CNS complications include cognitive impairment (26), seizures (27, 28), cranial neuropathy (29, 30), extrapyramidal disorders such as Parkinson’s disease (31) and chorea (32). Pediatric patients are more prone to the CNS damage caused by uremia, including microcephaly, retardation, cerebral edema, recurrent seizures and ataxia (9–11). Moreover, Hurkx et al. found delayed peak of brainstem auditory evoked potentials and unusual sensory evoked potentials in younger children, suggesting unusual myelination and synapse formation in children with chronic renal failure (CRF) (39).

**Table 1:** The symptoms of uremic neuropathy.

Category	Symptoms	Reference
central nervous system	cognitive impairment	(26)
	seizures	(27, 28)
	cranial neuropathy	(29, 30)
	extrapyramidal disorders	(31, 32)
	microcephaly	(9–11)
	retardation	(9–11)
	cerebral edema	(9–11)
	cerebral edema	(9–11)
	hearing impairment	(12)
	cerebral palsy intellectual disability	(13)
peripheral nervous system	loss of tendon reflexes	(33)
	muscle atrophy	(33)
autonomic nervous system	sweat glands dysfunction	(34)
	valsalva maneuver disorder	(35)
	reduced baroreflex sensitivity	(36)
	hypoesthesia	(37)
mental changes	emotional changes	(28)
	disturbing	(28)
	depression	(38)

The symptoms of peripheral neuropathy include loss of tendon reflexes and muscle atrophy (33). The ANS complications is manifested by defective function of sweat glands (34), abnormal response to the valsalva maneuver (35), decreased baroreflex sensitivity (36), and hypotension (37). Psychological abnormalities include depression emotional changes, disturbing and depression (28, 38). Thus, the symptoms of uremic neuropathy are very complex and lack clear clinical, laboratory, or radiographic findings. The diagnosis of uremic neuropathy is frequent retrospective, only when symptoms improve after dialysis or kidney transplantation as well as other causes of neuropathy have been excluded (40). Although the prevalence of uremic neuropathy in children is not available, it has been reported it is quite common in children with CKD on maintenance hemodialysis (25). Neurologic complications contribute largely to the disability and mortality in patients with kidney failure. The age of onset and duration of CDK may influence neurocognitive and functional outcomes (41). Early life is an important period for the rapid proliferation and myelination of glial cells, and any significant insult can have enduring effects on neural structure and function (42, 43). Further knowledge of the potential link between uremia and neurological symptoms is needed, especially in children, to better understand this complication and to exert early intervention.

### 3.2 The general underlying mechanisms of uremic neuropathy

#### 3.2.1 Uremic toxin accumulation

The development of uremic syndrome is usually caused by the retention of retained solutes that are filtered by healthy kidneys and have deleterious effects on biological function, known as uremic toxins (44). The European uremic toxin working group proposed a classification of 90 uremic solutes in 2003 (2). By reviewing the literature, Flore et al. identified 88 uremic toxins (45). According to their physicochemical properties, uremic toxins can be classified into small water-soluble compound, middle molecular compounds and protein-bound compounds (**Table 2**). Due to the difference in hydrophobicity, small molecular weight uremic toxins can exist as a free water-soluble form or reversibly bind to serum proteins, thereby altering protein function (75). Several studies suggest that these toxins may originate from diet or microbial metabolism as they can also be produced in the gut (76, 77). Recently, mitochondria have been implicated as contributors to uremic toxin production. Mitochondria may directly impact the synthesis of uremic toxins, especially the oxidation products or peroxidation products of cellular components, and uremic toxins in turn can cause damage to mitochondria, thereby producing more uremic toxins, forming a positive feedback loop that leads to increased production of uremic toxins (78). Studies also have reported that uremic toxins contribute to uremic neuropathy (79). Understanding the relationship between uremic toxins and neurological disorders contribute to better comprehend the pathogenesis of uremic neuropathy.

**Table 2:** The uremic toxins affecting neurogoical disorder.

Category	Uremic toxins	Neurogoical disorder	Pathologicalmechanisms	Reference
small water-soluble compounds	guanidine compounds	·cognitive dysfunction ·neuronal damage ·neurodegeneration	·oxidative stress ·block gamma aminobutyric acid and glycine receptor-associated ion channels ·enhance the pro-apoptotic effects of H_2_O_2_ and altering mitochondrial calcium homeostasis in glial cells	(46–53)
	hypoxanthine	·neurological damage	·elevated levels of free radicals and uric acid	(54–58)
middle moleculars	parathyroid hormone	·reduce the conduction velocity of motor nerve ·interfere with nerve transmission ·cognitive dysfunction	·increase calcium entry into the brain	(56–64)
	β2-microglobulin	·impair hippocampal cognition and neurogenesis	·neurotoxicity ·pro-aging	(65, 66)
Protein-bound solutes	indoxyl sulfate	·Parkinson’s disease ·emotional disturbance ·neuroinflammation	·expression of organic anion transporter 3 (OAT3) efflux transporter ·oxidative stress ·protein kinase inhibition	(67–70)
	homocysteine	·dementia	·activating the glutamate N-methyl-d-aspartate (NMDA) receptors	(71–74)

##### 3.2.1.1 Small water-soluble compounds

###### 3.2.1.1.1 Guanidine compounds

Guanidine compounds (GCs), such as guanidinosuccinic acid (GSA), guanidine and methylguanidine, are considered to be the major cause of cognitive dysfunction in uremic syndrome of CKD (46–48). The accumulation of guanidine compounds can trigger oxidative stress (49) and lead to neuronal damage (50). Study in uremic animal models reported that GSA impaired synaptic function in CA1 region and affected memory process by blocking gamma aminobutyric acid (GABA) and glycine receptor-associated ion channels (51). It was also found an elevation of GSA and methylguanidine in the epileptic brain in animal models (52). Moreover, methylguanidine may promote uremia-related neurodegeneration by enhancing the pro-apoptotic effects of H_2_O_2_ and altering mitochondrial calcium homeostasis in glial cells (53).

###### 3.2.1.1.2 Hypoxanthine

Hypoxanthine, a metabolite of purines, has been shown to have effects on the CNS (54). Hypoxanthine damages rat hippocampus and striatum. These effects are mediated by elevated levels of free radicals and uric acid, which lead to changes in acetylcholinesterase and butyrylcholinesterase activity (55). Hypoxanthine can be converted to xanthine by xanthine oxidase (XO) and subsequently to uric acid and have been shown to be positively associated with lipid peroxidation (56). Consistent with this result, XO inhibition ameliorates brain injury and renal dysfunction (57, 58).

##### 3.2.1.2 Middle molecular compounds

###### 3.2.1.2.1 Parathyroid hormone

Excessive parathyroid hormone (PTH) is mainly caused by hyperparathyroidism due to hyperphosphatemia that occurs in CDK (59, 60). Elevated parathyroid hormone reduces the conduction velocity of motor nerve in uremic patients (79). Hyperparathyroidism increases calcium entry into the brain and interferes with nerve transmission, thereby inducing neurotoxicity (61), while removal of parathyroid glands can prevent calcium excess in uremic brain (62). Furthermore, parathyroidectomy can prevent electroencephalogram (EEG) abnormalities in uremic animals, whereas administration of parathyroid extract to normal animals can induces similar EEG changes to uremic animals (63). It has also been suggested a harmful effect of PTH on cognitive function in CKD patients (64). However, there is still a lack of research supporting this view, which needs further investigation.

###### 3.2.1.2.2 β2-microglobulin

Limited information is available on the relationship between β2-microglobulin and neuropathy. It has been reported that β2-microglobulin is cytotoxic for the SH-SY5Y neuroblastoma cells at concentrations readily attainable in the plasma of hemodialysis patients (65). Despite the potential neurotoxicity of β2-microglobulin, the concentration of β2-microglobulin in CSF is maintained at a low level due to the protective effect of the blood-brain barrier (BBB). However, BBB may be damaged in uremic patients. Furthermore, β2-microglobulin has been also identified as a pro-aging factor that impairs hippocampal cognition and neurogenesis (66).

##### 3.2.1.3 Protein-bound compounds

###### 3.2.1.3.1 Indoxyl sulfate

The serum indoxyl sulfate concentration in uremic patients was markedly increased (80). The average serum level of total indoxyl sulfate in patients with uremia was approximately 43 times higher than that in normal subjects (45). The accumulation of indoxyl sulfate within brain structures may be related to the expression of organic anion transporter 3 (OAT3) efflux transporter (67). Indoxyl sulfate involve in human astrocyte apoptosis through oxidative stress and protein kinase inhibition (68). Bartłomiej et al. also reported that the level of indoxyl sulfate in the cerebrospinal fluid of patients with Parkinson’s disease was higher than expected, which may affect the progression of Parkinson’s disease (69). Furthermore, intraperitoneal injection of indoxyl sulfate leads to emotional disturbance in mice unilateral nephrectomy, which is associated with altered neuronal cell function and neuroinflammation (70).

###### 3.2.1.3.2 Homocysteine

The serum level of total homocysteine in uremic patients was significantly higher than that in the healthy people (81). Homocysteine has been demonstrated to display direct neurotoxic effects (71). Indeed, homocysteine causes direct neurotoxicity in cortical neurons by activating the glutamate N-methyl-d-aspartate (NMDA) receptors (72). Overactivation of these receptors may mediate ischemic damage in the brain (73). Thus, homocysteine may be involved not only in cerebrovascular injury, but also in neurotoxicity. A prospective cohort study reported plasma homocysteine as an independent risk factor that may play a persistent role in dementia (74). Further studies are needed to assess the impact of hyperhomocysteinemia in uremia-related neurological damage.

#### 3.2.2 Oxidative stress

Oxidative stress can be regarded as an imbalance between reactive oxygen species (ROS) production and antioxidant defense. This disturbance can impact cellular function and damage nucleic acids, proteins and lipids (82). Numerous studies have demonstrated increased oxidative stress in uremic patients. Low-density lipoproteins (LDL) from uremic patients are more prone to copper-induced lipid peroxidation than plasma LDL from healthy subjects *in vitro* (83). In addition to lipid peroxidation, uremic oxidative stress is also biochemically characterized by a state of accumulation of reactive aldehydes and oxidative thiols and depletion of reductive thiols (84). Reactive aldehydes can be regarded as end products of various oxidation reactions, such as the oxidation of alcohol and amino (85). Several reactive aldehyde compounds, including acrolein, malondialdehyde, methylglyoxal, glyoxal, and hydroxynonenal have been demonstrated to be 10-fold higher concentrations in uremic plasma than that in healthy individuals (86, 87). In particular, α, β-unsaturated aldehydes are important oxidation products with capable of forming advanced glycation end products (AGEs) (88). In uremia, the accumulation of reactive aldehydes is primarily due to reduced renal catabolism and increased production by myeloperoxidase-catalyzed activation of phagocytes (84). Thiols, important antioxidant component, are found to be depleted in hemodialysis patients and thus is unavailable to participate in antioxidant defense (87). Furthermore, plasma glutathione levels decreased in hemodialysis patients and accompanied by a significant decrease in glutathione peroxidase function (89). Mitochondria are also involved in the production of ROS. Impaired mitochondrial respiratory system in CKD patients is deemed as a consequence of enhanced oxidative stress, which may explain the abnormal energy metabolism in this population. Many uremic toxins such as indoxyl sulfate, p-cresyl sulfate, kynurenic acid and hippurate can inhibit the mitochondrial respiratory chain and lead to the overproduction of ROS (78).

##### 3.2.2.1 The role of oxidative stress in uremic neuropathy

Neuronal tissue is quite vulnerable to oxidative stress (90). Oxidative stress not only causes direct neurotoxicity through phospholipid membrane peroxidation (91), but also incites excitotoxicity by promoting glutamate release (92, 93). This leads to the activation of NMDA and non-NMDA receptors, resulting in severely elevated intracellular Ca^2+^ levels, neuronal nitric oxide synthase (NOS) activation, peroxynitrite formation, protein nitration, mitochondrial damage, and ultimately causing neuronal damage or death (84). As mentioned above, lipid peroxidation, increased AGEs, and mitochondrial dysfunction are present in uremic patients. Lipid peroxidation can cause several deleterious effects during the development of neurodegenerative diseases (94). Since lipid peroxidation can increase the production of N-carboxymethyl lysine, which is a major form of AGEs (95). It has reported that the expression of AGEs receptors increased in human peripheral neuropathy (96). AGEs induce hypertrophy of the endoneurial microvascular basement membrane by stimulating pericytes to release TGF-β and vascular endothelial growth factor (VEGF), thereby destroying the BBB (97). In addition, AGEs exert deleterious effects on cells by upregulating pro-inflammatory cytokines and triggering inflammation (98). Similarly, reduced energy due to mitochondrial dysfunction can lead to neuronal damage, death and neuropathy (99). Additionally, oxidative stress is associated with an increased inflammatory response. Indeed, oxidative stress and inflammation can form a feed-forward cycle, leading to neurodegeneration (100). Oxidative stress can directly or indirectly induce the production of pro-inflammatory cytokines by activating tumor necrosis factor-α (TNF-α) and nuclear factor-kappaB (NF-κB). These factors can trigger the inflammatory mechanisms in neurodegeneration (100, 101). As a stimulator, oxidative stress can also cause the dysfunction of proteasome, thereby enhancing free radical generation and protein oxidation, as well as inhibiting ubiquitination. Consequently, unwanted proteins will accumulate in the cytoplasm and induce the formation of senile plaques (102), which is closely related to cognitive impairment and Alzheimer’s disease (103).

#### 3.2.3 Neurotransmitter imbalance

Neurotransmitters are defined as substances that carry messages between neurons. When neurotransmitters bind to neurotransmitter receptors, it can transmit either excitatory or inhibitory signals, thereby triggering a series of responses. However, when neurotransmitters become dysregulated, it can result in neurological disorders (104). Franz et al. found that both the extracellular concentrations of neuroexcitatory and neuroinhibitory amino acids increased in medial preoptic area (MPOA) of chronic renal failure rats (105). Moreover, Schaefer et al. found that basal outflow of synthetic g-aminobutyric acid (GABA), glutamate and aspartate was increased in the uremic rat brain (105). Consistent with this result, Biasoli et al. demonstrated altered transport of amino acids across the BBB and resulted in a high phenylalanine-tyrosine ratio and low glutamine level in the brain (106). Similarly, some other researchers found the phenomenon of plasma/cerebrospinal fluid amino acid imbalance in infants with CRF. The accumulation of abnormal amino acids in CSF was detrimental to the function of Na-K pump in synaptosomes, and might affect the development of neurons and astroglia, which may partially explain the potential mechanisms of impairments caused by CRF in pediatric patients (107).

Glutamine is the main source of synthetic GABA, and low levels of glutamine in the brain suggest the deficiency of GABA (108). Perry et al. dissected 10 patients with ESRD and dialysis encephalopathy and found decreased GABA levels in many brain regions, with reductions of more than 40% in the cortex and thalamus. Clinical consequences of GABA deficiency may be associate with cognitive impairment (109). This may suggest that cognitive impairment in uremia may be related with reduced GABA. As a well-known neurotransmitter, acetylcholine plays a multifaceted role (110). However, renal failure results in a disorder of acetylcholine metabolism, which leads to its accumulation and release in brain synaptosomes. This is mainly due to decreased activity of choline kinase, to some extent, mediated by the secondary hyperparathyroidism of renal failure (111). Thus, behavioral and motor changes in uremic patients may be due in part to disturbances in acetylcholine metabolism.

#### 3.2.4 Blood brain barrier disruption

BBB is a highly complex and dynamic structure involving endothelial cells, basement membranes, astrocyte foot processes, and pericytes that separates the CNS from the peripheral blood circulation (112). The BBB can tightly regulate the exchange of substances such as molecules and ions between the blood and the CNS. This is critical for strictly controlling the CNS homeostasis, as well as protecting the CNS from pathogens, inflammation, toxins, injury and disease (113). Recent studies have shown that BBB disruption play an essential role in neurodegenerative processes (114, 115). Meanwhile, some animal models of acute and chronic renal failure show BBB disruption in the context of uremia, but the mechanism remains unclear (116, 117). It has been reported that uremia increases the permeability of BBB as assessed by radioisotopes (118). Some uremic toxins, such as indoxyl sulfate, has been reported to mediate the activation of the transcription factor aryl hydrocarbon receptor (AhR) to induce BBB disruption and uremia-related cognitive impairment. Uremia activates inflammatory and oxidative pathways and inhibits antioxidant and cytoprotective systems, which erode the brain’s capillary-junction complexes (119). This suggests a possible disruption of the integrity of the blood-brain barrier in uremic patients.

### 3.3 The alteration of neuroimmune

Recent studies have uncovered the interaction between the nervous and immune systems, which can modulate immune function and inflammation, known as neuroimmune (120). One of the well- explored neuroimmune pathways is the cholinergic anti-inflammatory pathway (CAP). Chemicals released in the inflammatory bind to receptors on sensory nerve terminals and transmit neuronal signals to the brain *via* the afferents of vagus (121). This signal activates the vagal efferent nerve through the nucleus of the solitary tract and the dorsal vagus motor nucleus. The activated efferent vagal nerve stimulates the splenic nerve, although whether there is a direct connection between the efferent nerve of vagus and the splenic nerve remains controversial (122). Subsequently, norepinephrine is released from splenic nerve terminals and bind to β2-adrenergic receptors expressed by CD4^+^T cells in the spleen to mediate acetylcholine release from these cells (123). Acetylcholine interacts with the α7 nicotinic acetylcholine receptors (α7nAChRs) expressed on macrophages, which are key mediators to the regulation of neuroimmune, thus leading to inhibition of NF-κB and activation of Janus kinase 2 signal transducer and activator of transcription 3 (JAK2-STAT3) pathway, and ultimately inhibiting the production of pro-inflammatory cytokines, such as TNFα, and suppressing inflammation (124–126). Notably, the neuroimmune cross-talk between the nervous system and the kidney to maintain a normal physiological state. However, pathological state, such as uremia, can disrupt this interaction, further leading to disturbances in homeostasis that may ultimately result in neuropathy (127).

#### 3.3.1 Inflammatory cytokines and cells

Multiple factors can contribute to immune dysregulation and inflammatory activation in uremic patients. Uremia exhibits a persistent low-grade inflammatory environment, involving impaired inflammatory cell function and dysregulated cytokine networks. Some of these factors may be related to the primary disease, and some may be due to the highly proinflammatory oxidative stress generated by the uremic environment (128). Indeed, a feed-forward loop is formed between oxidative stress and inflammation, which lead to neurodegeneration (100). Oxidative stress can induce production of pro-inflammatory cytokines directly or indirectly *via* activating TNF-α and NF-κB (129–131). Available data suggest that the anti-inflammatory cytokine interleukin-10 (IL-10) and the major pro-inflammatory cytokines IL-6 (IL-6) and TNF-α may play critical roles in uremia (132). The cerebrorenal interactions suggested that IL-6 and TNF-α are likely to have an effect on the CNS, although the mechanisms are yet to be identified (133). NALP3, the best characterized inflammasome, act as a connection between inflammation and immunity. The NALP3 is activated in immune peripheral cells isolated from uremic patients. These cells show higher levels of mRNA of NALP3, IL-1β and IL-18 compared to healthy subjects (134). Interestingly, metabolic acidosis is another cause of inflammation among chronic hemodialysis patients, which develop with a decrease in glomerular filtration rate (GFR) (135, 136). Uremic toxins may contribute to gut dysbiosis in CKD and facilitate the translocation of gut bacteria, which in turn activates systemic inflammatory responses (137). Furthermore, protein-bound uremic toxins, such as indoxyl sulfate exert a proinflammatory effect by stimulating crosstalk between leukocytes and blood vessels, resulting in vascular injury (138). In addition, leukocyte oxidative burst, that is, the production of large amounts of ROS *via* the nicotinamide adenine dinucleotide phosphate (NADPH) oxidase complex (139), may further aggravate the inflammatory response (140, 141).

#### 3.3.2 Immune cells

##### 3.3.2.1 Innate immune system

###### 3.3.2.1.1 Polymorphonuclear neutrophils

Polymorphonuclear neutrophils (PMNLs), named after their lobulated nuclei, contain multiple granules in their cytoplasm and are particularly important in nonspecific cellular immunity (142). In patients with ESRD, the expression of toll-like receptor 2 (TLR2), TLR4 and integrins on PMNLs is increased (143). This increased expression is a key mediator of oxidative stress and inflammation associated with renal failure, which contribute to tissue damage in this population (144). The level of ROS production, degranulation, and extracellular trap formation in PMNLs from dialysis patients are significantly elevated, suggesting spontaneous activation (145). Synergistic elimination of activated PMNLs is critical for resolution of inflammation (146).

###### 3.3.2.1.2 Monocytes and macrophages

Monocytes are produced in the bone marrow and distributed in all body tissues as macrophages (147). Circulating monocytes were widely expanded in ESRD patients, especially CD14^+^CD16^+^ subsets (143). This may be due to the increased expression of integrins, TLRs and proinflammatory cytokines in uremic condition. The possibility therefore might lead to the release of CD14^+^CD16^+^ monocytes from the bone marrow (148). These abnormalities suggest spontaneous activation of monocytes and contribute to the prevailing oxidative stress and inflammation in ESRD. Thus, the number of circulating monocytes in ESRD patients, particularly the CD14^+^CD16^+^ subsets might be related to the degree of systemic inflammation and/or uremia.

###### 3.3.2.1.3 Dendritic cells

Dendritic cells are considered to be the major professional antigen presenting cell in the immune system (149). Uremia has a negative effect on dendritic cells. Study have shown that monocyte-derived dendritic cells from CKD patients are less able to stimulate T cells than healthy controls (150). This may be related to the decreased expression of the key costimulatory molecule CD86 in the uremic situation (151). Dendritic cells are also present in the renal and involve in the progression of kidney failure by binding to glomerular antigens (152).

##### 3.3.2.2 Adaptive immune cells

###### 3.3.2.2.1 T cells

Exposure of naive T cells to antigen leads to clonal expansion and differentiation of memory T cells and effector T cells, which play a central role in cell-mediated immune responses (152). Patients with ESRD showed significantly reduced naïve and CD4^+^ and CD8^+^ T cells (153). Furthermore, the proliferation of T cell in ESRD patients have been reported to be impaired when detect in uremic serum (154). This is associated with increased sensitivity to apoptosis in both naive and memory T cells from uremic patients (155).

###### 3.3.2.2.2 B cells

B lymphocytes are mainly produced by hematopoietic stem cells in the bone marrow and mediate humoral immunity by producing antigen-specific antibodies (156). Similar to T cells, B cell lymphopenia in ESRD patients is associated with progressive loss of renal function (157). Additionally, CD5+ innate B cells and CD27^+^ memory B cells are reduced in childhood chronic renal failure (158). Fernández et al. reported that uremic environment may sensitize B cells to apoptosis in ESRD patients (159). Madeleine et al. reported that the uremic environment may interfere with the differentiation and survival of B cells by down-regulating the BAFF receptor in transitional B cells in ESRD patients (157). Thus, the deficiency and dysfunction of B cells in advanced CKD may be mediated simultaneously by increased B cell apoptosis and impaired differentiation and maturation of transitional B cells.

## 4 Potential treatment for uremia

Hemodialysis and renal transplant are two common treatments for uremia. Efficient solute removal usually improves CNS symptoms over multiple dialysis sessions. However, dialysis is relatively effective only for some small water-soluble solutes, and some protein-bound solutes cannot be effectively removed. Kidney transplants are also effective at removing uremic toxins. Patients with kidney transplant are given immunosuppressants and prophylactic antibiotics, which cause changes in the gut microbiota (160).

### 4.1 Anti-inflammatory therapy

As mentioned above, pro-inflammatory factors play an important role in uremia, therefore, targeted anti-inflammatory therapy is recommended for these patients. Statins, a typical lipid-lowering drug, has been demonstrated to have an anti-inflammatory effect in hemodialysis patients (161). Angiotensin-converting enzyme (ACE) inhibitors can suppress TNF-α in advanced chronic renal (162). In patients with chronically uremia, pentoxifylline was confirmed to reduce the whole-body proteolysis, a characteristic of systemic inflammation activation (163).

### 4.2 Neuroimmunomodulatory therapy

Besides, neuroimmunomodulation of organ function has attracted extensive attention as a new treatment for diseases. Considering the anti-inflammatory effect of CAP pathway, stimulation of vagal nerve, has been shown to protect the kidney from AKI (164). However, electrical stimulation of the vagus nerve requires invasive surgery. In clinical application, ultrasound, a non-invasive imaging technique, can be used as a candidate for the activation of CAGigliotti et al. reported that ultrasound can suppresses inflammation in renal by stimulating the CAP pathway (165). This result might lead to a powerful nonpharmacological approach to block the inflammation of kidney and preserve function. Further studies are needed to explore the underlying mechanisms of neuroimmunomodulatory in inflammation and organ damage.

## 5 Limitations of current researches and perspectives

The paucity of data on pediatric uremia is a thorny problem. Due to the differences in region, culture and detection methods, the heterogeneity of the existing data is relatively high. For this reason, we discuss a general underlying mechanism of uremia. Therefore, a unified standard is urgently needed to register and follow up these patients. Besides, the association between uremic toxins and uremic neuropathy has not been fully elucidated, especially in children, which may require large prospective studies. Finding biostatistical associations between specific clinical signs and solute concentrations by combining high-throughput analytical methods with clinical databases represents an intriguing but underexplored track to unravel the pathophysiology of uremic neuropathy.

Moreover, the roles of immune cells and inflammatory factors in the uremic immune microenvironment and the inflammatory microenvironment remain to be explored, and the temporal and spatial distribution of immune cells and inflammatory factors needs to be further investigated. New methods such as single-cell sequencing and temporal-spatial omics need to be used to further explore the underlying mechanisms. In addition, non-invasive treatments, neuroimmune interventions, and targeted anti-inflammatory treatments in uremia, such as dose gradients or time gradients, need to be further explored.

Going forward, technological innovations and the combined efforts of the global scientific community may help elucidate some fundamental questions about the causes and treatment of uremic neuropathy in children, and provide new insights for this disease that can help pediatricians prescribe tailored treatment for children.

## 6 Conclusion

**Figure 1:**
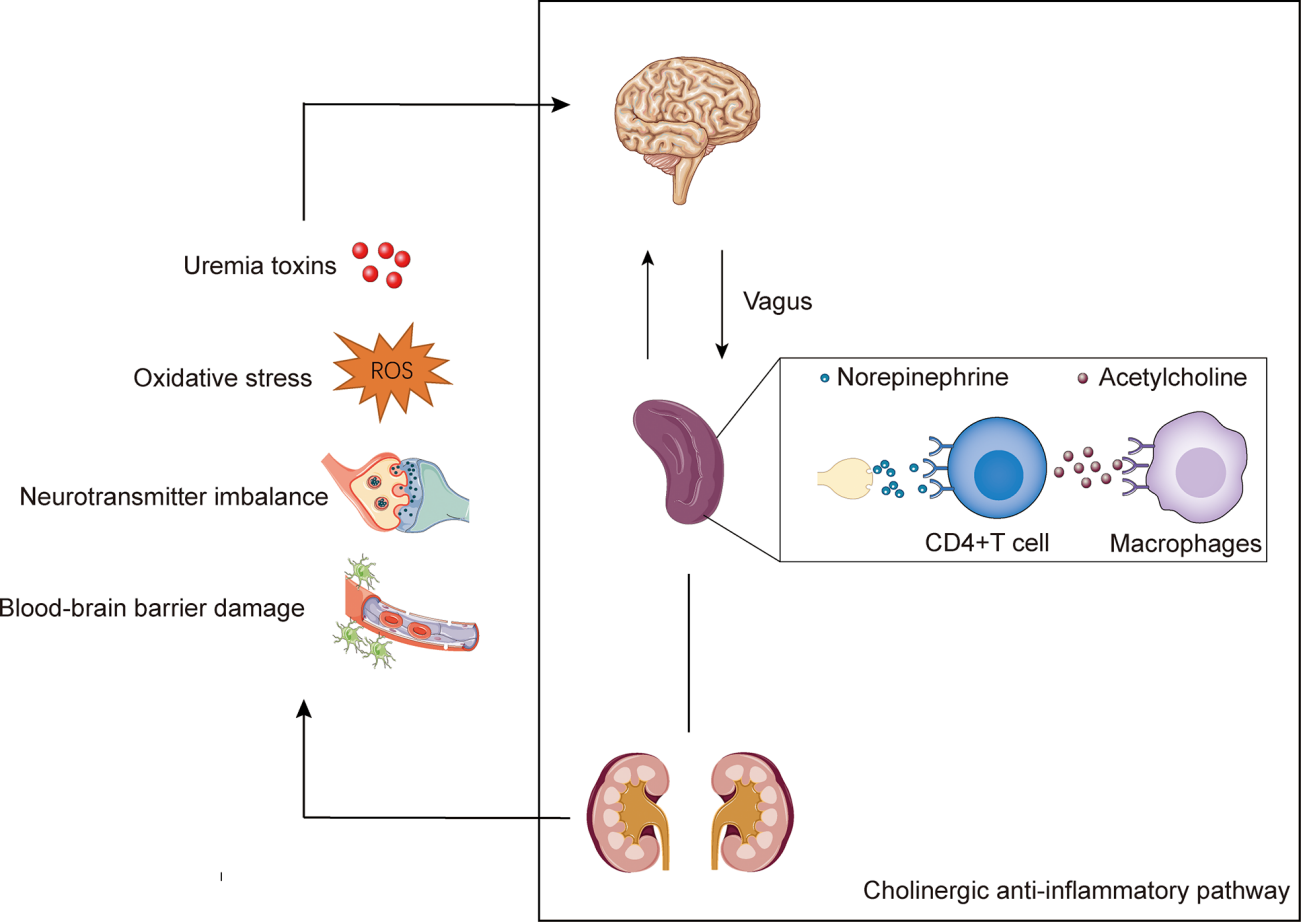
The potential mechanisms of uremic neuropathy and the cholinergic anti-inflammatory pathway. Uremic neuropathy is associated with accumulation of toxins, oxidative stress, neurotransmitter imbalance, and disturbance of the blood-brain barrier. These factors may lead to the release of inflammatory factors and stimulate the sensory afferent vagus nerve endings. The cholinergic anti-inflammatory pathway is starting with vagal activation. Signals transmits to the brain *via* the afferents of vagus. The activated efferent vagal nerve stimulates the splenic nerve and release norepinephrine. The norepinephrine binds to β2-adrenergic receptors expressed by CD4^+^T cells in the spleen and mediate the of release acetylcholine. Acetylcholine interacts with the α7 nicotinic acetylcholine receptors (α7nAChRs) expressed on macrophages, and ultimately inhibiting the production of pro-inflammatory.

Pediatric uremic neuropathy is an extremely complex complication that leads to morbidity and mortality in children. In this review, we discuss the epidemiology of childhood uremic neuropathy, the underlying mechanisms, possible treatments, and current research gaps (**Figure 1**). Uremic neuropathy is associated with accumulation of toxins, oxidative stress, neurotransmitter imbalance, and disturbance of the blood-brain barrier. Among them, neuroimmune, including the change of inflammatory factors and immune cells, play a crucial role in the progression of uremic neuropathy. Different from traditional dialysis and kidney transplantation, treatments that focus on anti-inflammation and neuroimmune intervention show promising prospects.

## Author contributions

LZ and GT wrote the main manuscript text. GC revised the manuscript. FY and YZ prepared figures and table. All authors contributed to the article and approved the submitted version.

## Funding

This study was funded by the Basic Public Welfare Research Project of Zhejiang Province (LGF22H050004) and Medical and Health Technology Program of Zhejiang Province (2022481900).

## Acknowledgments

We thank all the authors of the original work and reviewers for their time and kindness in reviewing this paper. The authors would like to thank servier medical art for providing open access to the picture materials.

## Conflict of interest

The authors declare that the research was conducted in the absence of any commercial or financial relationships that could be construed as a potential conflict of interest.

## Publisher’s note

All claims expressed in this article are solely those of the authors and do not necessarily represent those of their affiliated organizations, or those of the publisher, the editors and the reviewers. Any product that may be evaluated in this article, or claim that may be made by its manufacturer, is not guaranteed or endorsed by the publisher.

## Abbreviations

ESRD, end-stage renal disease; CDK, chronic kidney disease; AKI, acute kidney injury; CNS, central nervous system; PNS, peripheral nervous system; ANS, autonomic nervous system; GCs, guanidine compounds; GSA, guanidinosuccinic acid; XO, xanthine oxidase; PTH, parathyroid hormone; EEG, electroencephalogram; BBB, blood-brain barrier; OTA3, organic anion transporter 3; NMDA, N-methyl-d-aspartat; ROS, reactive oxygen species; LDL, low-density lipoproteins; AGES, advanced glycation end products; NOS, nitric oxide synthase; MPOA, medial preoptic area; GABA, gaminobutyric acid; AhR, aryl hydrocarbon receptor; CAP, cholinergic anti-inflammatory pathway; α7nAChRs, α7 nicotinic acetylcholine receptors; JAK2-STAT3, Janus kinase 2 signal transducer and activator of transcription 3; NF-κB, nuclear factor-kappaB; TNF-α, tumor necrosis factor-α; GFR, glomerular filtration rate; PMNLs, polymorphonuclear leukocytes; TLR2, toll-like receptor; ACE, angiotensin-converting enzyme.
